# Super-Resolution Imaging of Molecular Emission Spectra and Single Molecule Spectral Fluctuations

**DOI:** 10.1371/journal.pone.0147506

**Published:** 2016-03-22

**Authors:** Michael J. Mlodzianoski, Nikki M. Curthoys, Mudalige S. Gunewardene, Sean Carter, Samuel T. Hess

**Affiliations:** Department of Physics and Astronomy, University of Maine, Orono, Maine, United States of America; Julius-Maximilians-University Würzburg, GERMANY

## Abstract

Localization microscopy can image nanoscale cellular details. To address biological questions, the ability to distinguish multiple molecular species simultaneously is invaluable. Here, we present a new version of fluorescence photoactivation localization microscopy (FPALM) which detects the emission spectrum of each localized molecule, and can quantify changes in emission spectrum of individual molecules over time. This information can allow for a dramatic increase in the number of different species simultaneously imaged in a sample, and can create super-resolution maps showing how single molecule emission spectra vary with position and time in a sample.

## Introduction

In conventional far-field microscopy, diffraction limits the resolution of structures smaller than half of the detection wavelength, or roughly ~250 nm for visible light. In the last decade, several localization-based super resolution microscopy (LM) techniques have circumvented this limit and improved resolution by more than a factor of ten.[1–4] The final image resolution depends on the localization precision and the density of localizations and typically is on the order of tens of nanometers.[1, 2, 5]

Many fundamental biological questions involve relationships between molecular species; many of the numerous biological applications of LM depend on the ability to distinguish two or more molecular species within a sample. Previously, simultaneous multi-species LM has largely depended on schemes which split detected fluorescence into two channels based on emission wavelength [6–9] with the identity of each molecule determined from the ratiometric comparison of intensities in each channel. Due to the broad emission spectra of many fluorescent species, the limited number of photons detected per molecule, and background noise, correct differentiation between multiple fluorescent species can be difficult. Misidentification (incorrectly reporting one species as another) can be mitigated by selecting only the brightest molecules for the final rendered image, but this can also limit the density of localized molecules, which partly determines image resolution.[1, 2, 5] Although recent experiments have successfully used such methods to distinguish multiple molecular species using fluorescent protein labels,[8] organic dyes,[7, 8, 10] or a combination of organic dyes and proteins,[11] methods based on intensity ratios do have some limitations. Binning all photons into one of two channels sacrifices much of the information contained in the emission spectrum of each molecule, intensity ratios are susceptible to fluorescence background and background noise, and separation of increasingly larger numbers of fluorescent species in a sample becomes increasingly difficult.

**Fig 1:**
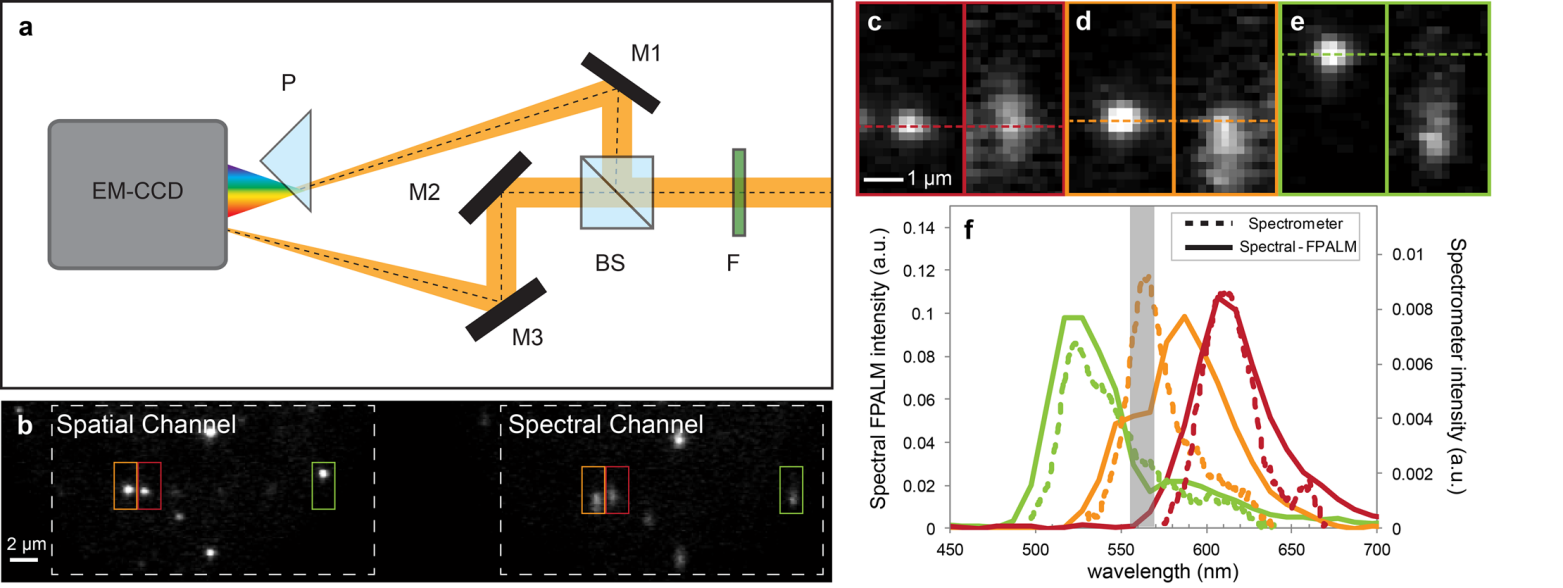
Spectral-FPALM experimental setup (a). Filters (F) blocked reflected laser light and cellular auto-fluorescence. Emitted fluorescence was split by a 50:50 beamsplitter (BS). The reflected path was redirected to the camera and spread into its spectrum with a dispersive prism (P) creating the Spectral Channel (b, right). Light transmitted through the BS formed the Spatial Channel (b, left). Two additional mirrors (M2 and M3) ensured that the same focal planes were imaged. Three different colors of fluorescent beads (yellow-green, orange, and red) were simultaneously imaged (b). Colored boxes represent spatial locations (b, Spatial Channel) and corresponding spectral images and spectral distributions (b, Spectral Channel). The red bead (c) was taken as the reference bead since there was no noticeable shift between the vertical positions in the spatial and spectral channels. The orange bead (d) and yellow-green bead (e) show downward shifts of the spectral distribution of a few pixels (d) to approximately 12 pixels (e), indicating different fluorescent spectra. Calibration enabled measurement of the spectral profile (f, solid lines) of each of the beads in (c, d, and e). The dip in spectral intensity ~560 nm (gray box) was due to notch filters. Measurements with a spectrometer (f, dashed lines) confirmed the spectral measurements of Spectral-FPALM. Scale bar is 2 μm in (b) and 1 μm in (c, d, and e).

Here, we expand on a method we have previously presented [12, 13], named Spectral-FPALM, which simultaneously measures both the spatial position and emission spectrum of individual fluorescent molecules. Rather than assigning each photon to either of two detection channels, the wavelength of each detected photon is determined to within a few to a few tens of nanometers. This enables determination of the peak emission wavelength and/or changes in the emission of each single molecule while simultaneously acquiring a super-resolution image. In Spectral-FPALM, detected fluorescence is divided into two paths by a 50:50 beamsplitter: one image records the positions of single molecules in the traditional FPALM approach, while fluorescence in the second path passes through a dispersive optic (prism), which spectrally spreads the molecular image, allowing the intensity as a function of wavelength to be measured (Fig 1). In contrast to other techniques,[6, 10, 13, 14] Spectral-FPALM directly measures the spectrum of each single molecule, enabling simultaneous imaging and identification of much larger numbers of probe species in one sample. Further, the method has relatively high detection efficiency and is compatible with photoactivatable and photoconvertible fluorescent protein labeling and live-cell imaging. Spectral FPALM enables an exciting new family of super-resolution imaging experiments which can report spectral emission changes due to pH, hydrophobicity, redox state, ion concentrations, temperature, or other factors, while also recording precise nanometer molecular locations.

## Materials and Methods

### Setup

Spectral-FPALM is a modification of the standard FPALM configuration.[1] Additional elements outside of the microscope are added to the detection path as shown in Fig 1. In order to minimize the detected background fluorescence, measurements were performed in a total internal reflection fluorescence (TIRF) excitation geometry. The activation laser was 405 nm (Crystalaser,Reno, NV) and the readout lasers were 556 nm (GCL-556-100, Crystalaser, Reno, NV) or 561 nm (Sapphire, Coherent, Santa Clara, CA). The filter cube contained the dichroic 561RU and bandpass 565LP (Semrock, Rochester, NY). Notch filters (405 nm and 561 nm Semrock, Rochester, NY) mounted in the detection path blocked back-reflected laser light. The fluorescence was recorded with an Andor iXon EM-CCD camera (DU-897 DCS-BV, Andor Technology, South Windsor, CT). In the detection path, the fluorescence was split with a standard 50:50 beam splitter cube (BS013, Thorlabs, Newton, NJ) with the transmitted fluorescence directed toward the camera by two mirrors to record spatial information. The reflected fluorescence passed through a dispersive prism (PS910, Thorlabs) placed at a distance of 2–8 cm in front of the camera to record spectral information. The additional mirrors in the transmitted path equalized the lengths of the transmitted and reflected light paths.

### Calibration and Analysis

Calibration between the spectral and spatial channels was performed by illuminating a 0.5 μm pinhole (Cat. No. 1–0.5, National Aperture, Salem, NH) with a monochromator (Ocean Optics, Dunedin, FL, Fig G in S1 File) to create a nearly diffraction limited spot imaged by the camera (Fig A in S1 File). Two parameters, the monochromator illumination wavelength and the lateral position of the pinhole, are adjusted to cover the entire relevant spectral range and spatial regions of interest for calibration purposes. The relevant spectral range depends on choice of fluorescent probe(s), dichroic mirror(s), and emission filter(s).

Analyses were performed using custom scripts written in MATLAB (Mathworks, Natick, MA). The calibration method combines all pinhole positions for each illumination wavelength (for example, 600 nm in Fig A in S1 File). The software then correlates the spatial channel to the spectral channel for each illumination wavelength. The entire set of correlations is then fit to a second order polynomial as a function of wavelength to determine the wavelength dependence of the correlation parameters.

To test the ability of Spectral-FPALM to separate multiple fluorescent species, bead samples were created using 40 nm latex beads labeled with yellow-green, orange, and red dyes (F8795, F8792, F8793, Molecular Probes, Eugene, OR). An eight chamber Nunc well (Thermo Fisher Scientific, Rochester, NY) was coated with poly-L-lysine (Sigma-Aldrich, St. Louis, MO) for 20 minutes and washed once with HPLC water (Fisher Scientific, Fair Lawn, NJ). Approximately 200 μL of 1:20000 diluted bead solution was placed in each chamber for 20 minutes, followed by one wash with HPLC water. The chamber was left to dry after the wash.

Bead samples were imaged with the Spectral-FPALM setup described above (Fig 1). Typical excitation intensities at the sample were ~0.15 kW/cm^2^ with the 405 nm laser. This value was carefully chosen so that the photon emission rates of the three different bead types were high enough to be adequately detected but not so high as to saturate the camera.

### Sample Preparation and Imaging

**Fig 2:**
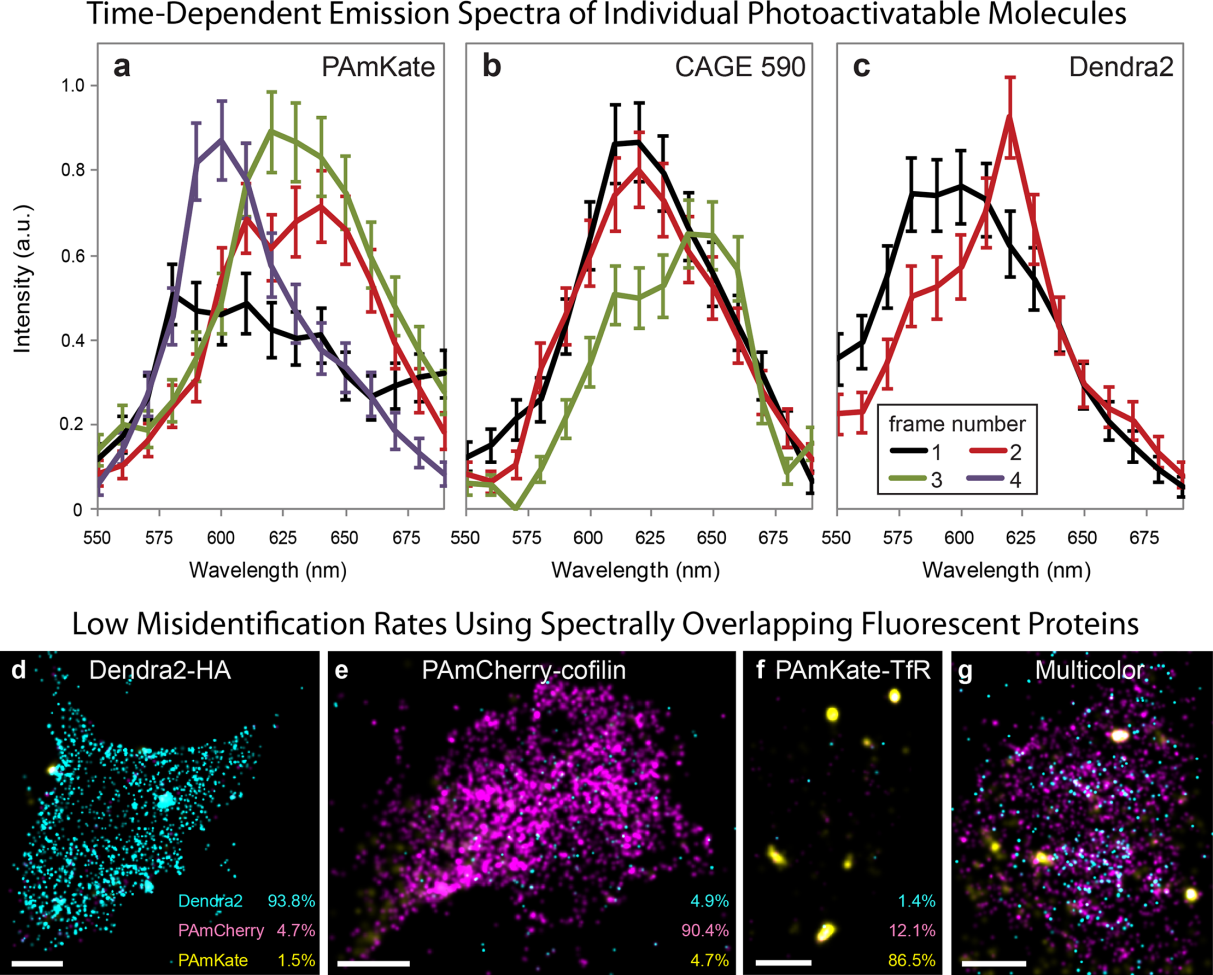
Spectral-FPALM measurements of spectral wandering and identification of multiple fluorescent species. Three examples of photoactivatable fluorophores undergoing spectral wandering are shown. Individual molecules spectrally wandered before photobleaching; (a) PAmKate, from mean emission wavelength ~600 nm to ~625 nm and back again; (b) CAGE 590 from ~620 nm to ~645 nm before photobleaching, and (c) Dendra2 from ~590nm to ~620nm before photobleaching. Error bars shown in (a-c) are due to shot noise from the number of detected photons. Single and multi- color images of NIH-3T3 cells were recorded using Spectral-FPALM. Single molecules were localized and identified based on the criteria shown in Fig B in S1 File. Each panel represents a different sample; Dendra2-HA (d), PAmCherry-cofilin (e), and PAmKate-TfR (f) and all three labels (g). The fraction of molecules identified as each fluorescent species is displayed at the bottom right of the single color cell panels (d-f). Misidentifications of Dendra2 (d) and PAmCherry (e) are less than 5%. Misidentification of PAmKate as PAmCherry is ~12%, largely due to fewer numbers of molecules in the PAmKate sample and large fraction of spectral wanderings of PAmKate (Table A in S1 File). Scale bars are 2 μm.

NIH-3T3 (ATCC, Manassas, VA) cells were transfected with Lipofectamine 2000 as per the manufacturer's protocol (Lipofectamine 2000, Invitrogen) with Dendra2-Hemagluttinin (HA)[15] or Dendra2-actin[16], PAmCherry-cofilin[17] or PAmCherry-tropomyosin4[16], or PAmKate-Transferin Receptor (TfR)[8]. For antibody labeling, NIH-3T3 cells were first fixed with 4% PFA. After permeabilization and blocking, the cells were incubated with a primary anti-mouse actin or anti-rabbit alpha-tubulin followed by an incubation with CAGE 552 goat anti-mouse IgG, CAGE 590 anti-rabbit IgG, or CAGE 635 anti-mouse IgG (Abberior, Göttingen, Germany) diluted 1:2000 in PBS. The samples were imaged with Spectral-FPALM as described. A complete set of experiments consists of calibration measurements (Fig A in S1 File), measurements of singly labeled cells for each probe in the experiment for species calibration purposes (Fig 2), and measurements of cells transfected/labeled with a combination of the fluorescent probes (Fig 2). Typically, between five and ten thousand frames were recorded at ~80–100 Hz and 200 EM gain for each data set. Laser intensities at the sample were ~8–12 kW/cm^2^ for 561 nm or 556 nm excitation and up to ~0.4 kW/cm^2^ of 405 nm activation.

Single molecules in both channels were localized as previously described.[1] Two methods of spectral measurement were performed. The localized position of the single molecule in the spatial channel was transformed (using the wavelength dependent calibration, Fig A in S1 File) to the spectral channel at defined wavelength intervals. The peak emission wavelength for each single molecule was determined by iteratively transforming the spatial position to the spectral channel until both the transformed spatial position and localized spectral position overlap. During the iterative transformation, the intensity of the spectral channel was recorded over the range of desired wavelengths in order to obtain the spectral distribution of the single molecule (Fig 2).

Samples of cells expressing one fluorescent probe provide a means of defining an acceptable range of (wavelength and intensity) values for identification of each fluorescent probe (Fig B in S1 File). If the boundaries are chosen carefully, low misidentification rates can be achieved (Fig 2).

### Spectral pixel size (SPS) as function of prism angle and distance from camera

We define the spectral pixel size (SPS) as the width of the wavelength range (in nanometers of emission spectrum) detected per camera pixel. The angle of fluorescence incident, *θ*, on the prism and the distance between the prism and the camera sensor, *d*, control the spectral pixel size (Fig C in S1 File). The angle of refraction of the fluorescence signal can be calculated by applying Snell’s Law to the fluorescence entering the prism and then applying Snell’s Law to the fluorescence exiting the prism. These angles and the distance to the camera were then used to determine the spectral pixel size. Fig C in S1 File shows the calculation of SPS as a function of fluorescence incidence angle, *θ*, and prism distance, *d*, from the camera (Fig C in S1 File). The open black circles (Fig C in S1 File) represent experimentally measured spectral pixel sizes at four different prism distances.

The spectral pixel size is also wavelength dependent due to the index of refraction having a non-linear relationship with respect to wavelength for the N-BK7 glass used to create the prism (PS910, Thorlabs, Newton, NJ). Fig D in S1 File shows the measured spectral pixel size as a function of wavelength for four different prism distances. Here, longer wavelengths disperse less than shorter wavelengths, resulting in a larger SPS at long wavelengths than at shorter wavelengths.

When imaging samples with high cellular autofluorescence (due to the cell type or when imaging in thick regions of the cell or in tissue), higher background noise can be counteracted by positioning the prism closer to the camera to reduce SPS while increasing the signal to noise. For probe identification, such an arrangement is less sensitive to uniform fluorescence background. At the other extreme, for applications with low background levels and/or with bright probes, the prism can be moved further from the camera in order to better disperse the spectra. This increases spectral resolution given that the signal-to-background ratio is high. The brightness, spectral distribution, and desired active molecule density of the probes must also be taken into account when deciding on the prism distance. With increased dispersion (low SPS), low-brightness probes may become difficult to identify against background and the emitter density must be lower in order to avoid overlap of the fluorescing single molecules. Bright probes such as CAGE dyes (Abberior, Göttingen, Germany), organic dyes, or quantum dots will still feature a high signal to noise when spread out over a large number of pixels.

## Results

To test the capabilities of Spectral-FPALM, we imaged three different colors of 40 nm fluorescent beads mixed within a single sample (Fig 1). Each bead color is readily identified by a spectral signature, as represented by the positional shift of the point spread function in the spectral channel relative to the spatial channel. Spectral- FPALM measurements (Fig 1, as described in the Materials and Methods section) of each bead spectrum in Fig 1 clearly show the three different bead colors. To confirm the Spectral-FPALM measurements, each of the different colors of beads shown in Fig 1 was measured using a spectrophotometer. Spectral-FPALM measurements match the spectrophotometer values (Fig 1), with a slightly larger width due to the contribution of diffraction to the Spectral-FPALM image.

Throughout the course of Spectral-FPALM measurements, we have observed the phenomenon of spectral wandering or spectral diffusion, originally observed in dyes at cryo[18, 19] and room[20, 21] temperatures as well as in quantum dots.[22] Measurements of the fluorescence emission of individual molecules of the photoactivatable protein PAmKate shifted during several consecutive image frames (Fig 2). In one case, the initial fluorescence in the first frame had a broad spectral distribution centered near ~600 nm, which is a much shorter peak wavelength than expected when compared to the bulk spectrum.[8] The fluorescence distribution then spectrally wandered so that the mean emission wavelength was ~625 nm for the second and third frames. In the final frame, the molecule spectrally wandered back toward the emission wavelength observed in the first frame. We have observed similar phenomena for all of the other photoactivatable probes studied (Dendra2 (Fig 2), PAmCherry (Fig F in S1 File), CAGE 552, CAGE 590 (Fig 2), and CAGE 635dyes). Table A in S1 File shows the fractions of photoactivatable proteins/dyes that spectrally wandered under typical measurement conditions; the longer that a single molecule fluoresces, the greater the probability of spectral wandering (Fig E in S1 File). To our knowledge, these were the first measurements to definitively measure spectral wandering of photoactivatable fluorescent proteins.

**Fig 3:**
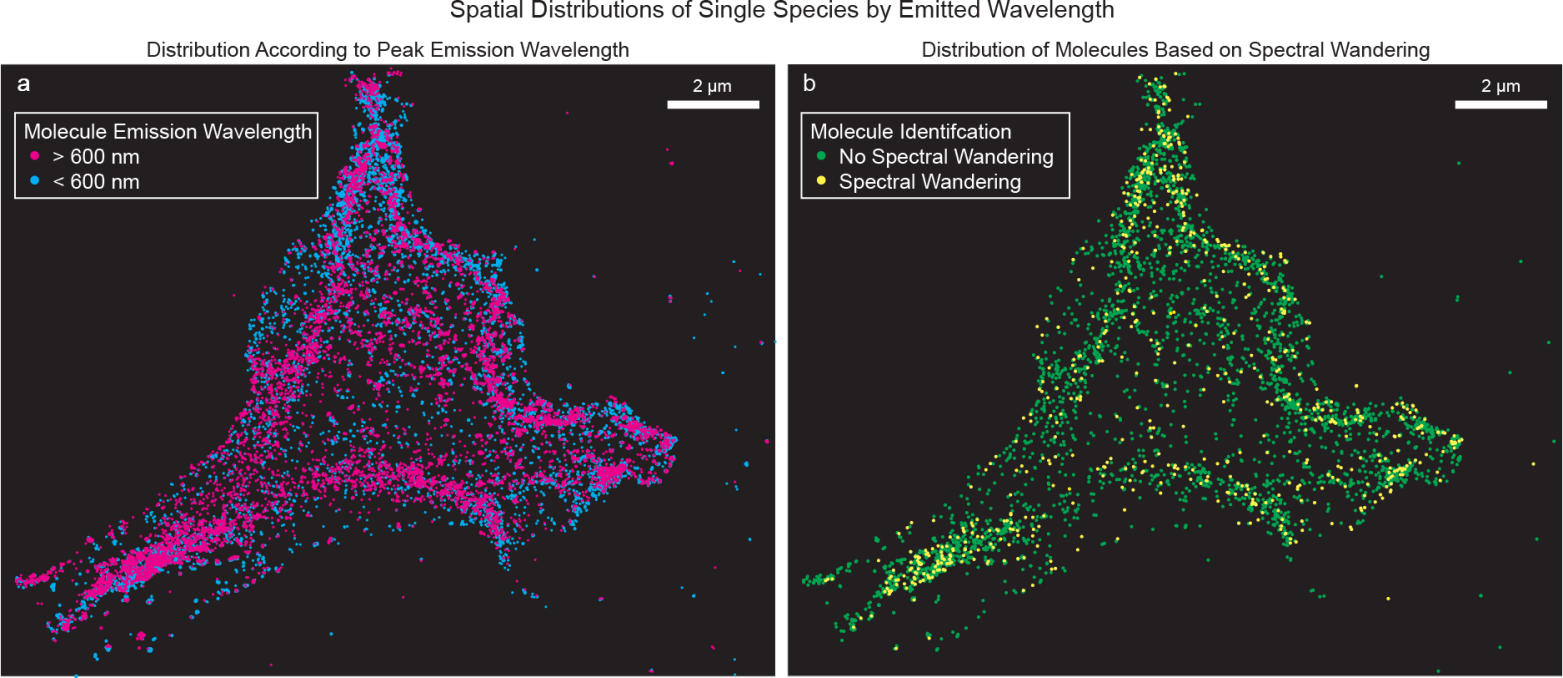
Super-resolution maps of spectral wandering and emission wavelength differences for localized individual molecules. (a) Super-resolution render of wavelength-dependence of emission spectra of individual localized molecules shows spatial gradients in emission wavelength of Dendra2-HA. Cyan points: molecules below the median wavelength. Magenta points: molecules above the median wavelength. Molecules which emitted for more than one consecutive frame are plotted using their position in each frame. Scale bar 2 μm. (b) Super-resolution render of spatial distribution of molecules with and without spectral wandering. Green points: molecules not exhibiting spectral wandering. Yellow points: molecules exhibiting spectral wandering. Molecules which emitted for more than one consecutive frame are each localized to an averaged single position. Scale bar 2 μm.

We demonstrate the use of Spectral-FPALM for multicolor LM in NIH-3T3 cells transfected with Dendra2-HA, PAmCherry-cofilin, and PAmKate-TfR. Localized molecular positions were determined from the spatial channel. The single molecules in the spectral channel were localized and the peak emission wavelength was determined (Fig A in S1 File). To help with differentiation between various fluorescent species, we were also able to incorporate the brightness[23] and localization precision of the single molecules (Fig 2 and Fig B in S1 File). While Spectral-FPALM can be used to perform multicolor super-resolution localization microscopy of large numbers of distinct fluorescent labels, it can also be used to observe variation in the emission spectra of a single species, and provide a super-resolution map of this variation. Fig 3 shows an example of spatial gradients in emission wavelength for a single fluorescent species. Differences in the emission wavelengths of fluorophores can result from changes in the state(s) of the molecules, which can be a function of the molecular environment.

Due to the observed spectral wandering of individual molecules and also to some extent due to the numbers of photons detected from each probe, the distribution of measured peak emission wavelengths had a FWHM of ~14–24 nm, depending on the probe species. Thus, the presence of spectral wandering can potentially complicate multi-species imaging, since it can cause probes which would on average emit distinctly to have, in some instances, similar emission patterns. However, spectral wandering also provides an opportunity in the case of Spectral-FPALM to create a super-resolution map of spectral fluctuations. Fig 3 shows a spatial map of localized molecules which showed (or did not show) spectral fluctuations (spectral wandering) over time.

## Discussion

Spectral-FPALM provides a viable and unique methodology for multicolor measurements. Other techniques using the ratiometric method are limited in their maximum number of fluorescent species simultaneously imaged, due to limited numbers of photons, shot noise, and background noise broadening the distribution of the intensity ratios. In addition, inherent spectral wandering of fluorescent probes will cause broadening of ratiometric distributions (especially for probes with emission spectra near the cutoff wavelength between channels) making identification of multiple species even more difficult. With Spectral-FPALM, the primary factors limiting the maximum number of fluorescent species are the detected spectral bandwidth, the detected background noise, and the emission spectra of the probes used. Setups which replace the traditional dichroic and bandpass filter cube with a small mirror[24] and only notch filters to prevent the activation and excitation lasers from reaching the camera can theoretically image over the entire detection wavelength range of the camera. Given a sample labeled with multiple photoactivatable/photoconvertable fluorophores, each separated in peak emission wavelength by ~20 nm, it is theoretically possible to simultaneously image in super-resolution up to 15–20 different fluorescent species, suggesting that this powerful technique could be harnessed to provide great leaps in understanding of many biological systems. At this point, however, generation of a sample labeled with such a large number of different fluorescent labels is non-trivial.

An additional strength of this technique is that the spatial channel provides the same information as in traditional LM. Using a single detection channel for spatial localization information eliminates potential complications from two channel calibration, as can occur in simultaneous multicolor imaging. While this method could be combined with a 4Pi geometry to further increase detection efficiency, the 4Pi geometry introduces additional geometrical constraints on the sample, and the single-objective method we present here provides sufficiently good signal-to-noise. Further, Spectral-FPALM is directly compatible with other imaging modalities, such as three dimensional imaging,[25, 26] anisotropy,[15] and live cell imaging,[27–29] using a variety of excitation configurations (e.g. TIRF, inclined illumination, widefield, 2-photon), in a wide variety of samples. A recent 2D particle tracking technique combining line scanning microscopy with spectral measurements of up to eight spectrally distinct quantum dot species has been published,[30] demonstrating full image readout of ~27 Hz and an ROI of ~28 μm^2^. In comparison, we present Spectral-FPALM data acquired at higher frame rates (80–100 Hz) with a larger ROI (>120 μm^2^). A greater increase in temporal resolution can be achieved by reducing the size of the ROI,[31, 32] or by using an even faster camera (i.e. an sCMOS). A further advantage of the additional spectral information is that it can potentially reveal the presence of two (spatially) overlapping fluorophores with distinct emission spectra. For example, where two molecules within 50 nm of each other would be otherwise indistinguishable and the localization algorithm would (erroneously) fit them as a single emitter, the spectral channel could potentially reveal that they are distinct molecules, providing additional a priori information to the localization algorithm [33, 34] to allow correct analysis of the image.

In addition to these extra capabilities which Spectral-FPALM allows for, we also here report the phenomenon of spectral wandering. These fluctuations of the emission spectrum of individual molecules of photoactivable proteins and dyes occurred on the order of tens to hundreds of milliseconds. This information, combined with the nanometer scale spatial localization, has allowed us to directly map the distributions of molecules, *of a single species*, according to their emitted wavelengths, and their spectral wandering. Such insight can itself be used to report a number of local, nanometer length scale fluctuations in molecular environmental conditions and probe states, so allowing the visualization of nano-compartmentalization within the cell. The power in such a capability may be further combined by describing the spectral wandering of a given probe with respect to a specific condition (such as pH), as allowing for a dramatic extra dimension of information to be yielded from a conventionally labeled sample.

## Supporting Information

S1 File The following file contains supplementary methods, equations, and figures. Figure A: Calibration Methods. Calibration images were created by imaging a 0.5 μm pinhole illuminated with specific wavelengths from a monochromator. A sample composite image of the calibration at 600 nm illumination is shown in (a). The pinhole is translated to several spatial locations, and the illumination through the pinhole at each location is recorded in the spatial channel (a, left) and spectral channel (a, right). The points in (a, left) represent nine different positions of the pinhole, with images from each position combined into one figure panel. Similar calibration images were created for each calibration wavelength (not shown). A cutout from the spatial channel (a, yellow box, left) and the corresponding spectral cutout (a, yellow box, right) are shown for illumination at 475 nm (spatial channel b, spectral channel c), 500 nm (d, e), 600 nm (f, g) and 700 nm (h, i). Dispersion translated the pinhole image in the spectral channel for different illumination wavelengths (c, e, g, and i). The intensity scale for (b-i) is the same as (a). Intensity variation was due to the light source, optical fiber transmission efficiency, and the camera efficiency. The scale bars are 2 μm (b) and 1 μm (c-j). Figure B: Determination of Identification Criteria using Single Species Images. Samples containing single species of Dendra2 (cyan points), PAmCherry (magenta points) or PAmKate (yellow points) are plotted together in graphs (a) and (b). The peak photon emission of each molecule was plotted against the localized wavelength for each molecule. (a) Each ellipse defines a fluorescent species, cyan for Dendra2, magenta for PAmCherry, and yellow for PAmKate. As magenta and yellow ellipses overlap, a second criterion (localization precision, plotted against wavelength, (b)) was used to separate molecules falling into both ellipses. For example, each point that falls within the yellow ellipse (a) and above the yellow line (b) was identified as PAmKate. Misidentified molecules were those points that appeared in the incorrect region (i.e. magenta points in the cyan ellipse (a)). Gray points did not satisfy any identification criteria. Figure C: Dependence of Spectral Pixel Size on Experimental Geometry. The fluorescence entrance angle, *θ*, at the prism (a) and the prism distance, *d*, from the camera (b) have a strong effect on the spectral pixel size. The curves were calculated by applying Snell’s law for the fluorescence signal as it enters and exits a right angle prism. The open black circles in (b) were experimentally measured values. Figure D: Spectral Pixel Size Measured as a Function of Wavelength and Distance Between Prism and Camera Sensor. Measured spectra cover larger numbers of pixels (i.e. smaller spectral pixel size, or SPS) when the prism is farther from the camera (solid black line) than when the prism is placed closer to the camera (short dashed line). Longer wavelengths of light expand less than shorter wavelengths due to the dependence of the index of refraction on wavelength. This results in larger spectral pixel sizes at longer wavelengths of imaged fluorescence. Figure E: The Probability of Spectral Wandering Increases with Time for the Fluorescent Probes under Investigation. The standard error of mean represents the error bars. Figure F: The Measured Spectra of PAmCherry Closely Matched the Spectra Measured with Spectral-FPALM. PAmCherry fluoresced for two camera frames (10.9 ms per frame) (small dashes) and underwent spectral wandering during the measurement. The two frames summed together (large dashes) closely approximate the measured bulk spectrum of PAmCherry. Figure G: Measurement of Spectra Generated with a Monochromator. A monochromator was used to produce light with peak emitted wavelength from 400 nm to 800 nm, at 50 nm intervals. This emission was then measured with a spectrometer. Peaks of each emission wavelength match the set wavelength to ±0.5 nm with a width of ~10–12 nm. Figure H: Theoretical Localization Precision vs. PSF. The localization precisions for a point spread function (PSF) and a PSF passed through a 0.5 μm pinhole were theoretically calculated as a function of wavelength. Twenty thousand PSFs were simulated and localized for each set of wavelengths. The width of the distribution of localized positions is taken as the localization precision (X markers) and is in good agreement with the theoretical calculations using the two dimensional Thompson localization equation.[33] The mean localization precision for the pinhole (O markers) is larger (worse) than the PSF localization values, as expected due to passing through the pinhole, but even in the worst case of 1000 photons, the localization precision is better than 12 nm. Experimentally, for the pinhole, N >5000 photons is easily achievable. This demonstrates that despite the pinhole being slightly larger than diffraction limited in size, its image can be sufficiently well localized to allow excellent alignment of the spectral and spatial channels, and to allow wavelength calibration of the spectral channel. Figure I: Calculated Localization Precision vs. Number of Detected Photons. The calculated localization precisions improved with a greater number of photons, as expected.[33, 34] The black line represents Eq. 1; the localization precision from Thompson et al 2002[33] expected for a single channel measurement. The red line represents Eq. 3, the localization precision expected when the number of photons is reduced (due to splitting into two channels) by a factor of *c*_*1*_ = 0.5. The green line represents Eq. 7 for the case when the total numbers of photons were split by *c*_*1*_ = 0.5, and the background level is split such that *c*_*2*_ = 0.4 (i.e. only 40% of background remains in that channel due to, for example, the wavelength dependence of the beamsplitter). For these calculations, *s* = 120 nm, *a* = 125 nm, and *b* = 3 photons. As expected, the localization precision is ~√2 worse when the number of photons contributing to the localization is reduced. Figure J: Emission of Mock-Transfected Cells. Spectral measurements conducted on mock-transfected NIH-3T3 cells with excitation of 561 nm and 405 nm at 4 kW/cm^2^ and 0.16 kW/cm^2^, respectively. Notch filters (405nm and 561nm) block the lasers from the spectrometer. Table A: Quantification of Emission Spectra of Individual Molecules Visible for Multiple Frames. Emission spectra shifted significantly for single molecules observed to fluoresce for multiple consecutive camera frames (10.9 ms/frame). The significance in this spectral wandering between frames was assessed with two-sample Kolmogorov-Smirnov tests, comparing the spectrum of one molecule in frame *n* with the spectrum of the same molecule in frame *n +1*. The average magnitude of the change in peak emission wavelength was recorded for each example of spectral wandering. The above data were calculated for molecules that fluoresced for two or more frames and contained 12 cells and 31,047 localizations from Dendra2; nine cells and 19,042 localizations from PAmCherry; six cells and 18,350 localizations from PAmKate; nine cells and 95,470 localizations from CAGE552; eight cells and 93,312 localizations from CAGE590; seven cell and 26,290 localizations from CAGE635.
(DOCX)
