# Supplementary material for: Super-Resolution Imaging of Molecular Emission Spectra and Single Molecule Spectral Fluctuations
Source: PLoS One. 2016 Mar 22;11(3):e0147506. doi: 10.1371/journal.pone.0147506 (PMC4803349; doi:10.1371/journal.pone.0147506)
Supplement: S1 File — Figure A: Calibration Methods. Calibration images were created by imaging a 0.5 μm pinhole illuminated with specific wavelengths from a monochromator. A sample composite image of the calibration at 600 nm illumination is shown in (a). The pinhole is translated to several spatial locations, and the illumination through the pinhole at each location is recorded in the spatial channel (a, left) and spectral channel (a, right). The points in (a, left) represent nine different positions of the pinhole, with images from each position combined into one figure panel. Similar calibration images were created for each calibration wavelength (not shown). A cutout from the spatial channel (a, yellow box, left) and the corresponding spectral cutout (a, yellow box, right) are shown for illumination at 475 nm (spatial channel b, spectral channel c), 500 nm (d, e), 600 nm (f, g) and 700 nm (h, i). Dispersion translated the pinhole image in the spectral channel for different illumination wavelengths (c, e, g, and i). The intensity scale for (b-i) is the same as (a). Intensity variation was due to the light source, optical fiber transmission efficiency, and the camera efficiency. The scale bars are 2 μm (b) and 1 μm (c-j). Figure B: Determination of Identification Criteria using Single Species Images. Samples containing single species of Dendra2 (cyan points), PAmCherry (magenta points) or PAmKate (yellow points) are plotted together in graphs (a) and (b). The peak photon emission of each molecule was plotted against the localized wavelength for each molecule. (a) Each ellipse defines a fluorescent species, cyan for Dendra2, magenta for PAmCherry, and yellow for PAmKate. As magenta and yellow ellipses overlap, a second criterion (localization precision, plotted against wavelength, (b)) was used to separate molecules falling into both ellipses. For example, each point that falls within the yellow ellipse (a) and above the yellow line (b) was identified as PAmKate. Misidentified molecule [file pone.0147506.s001.docx]

**Supporting Information**

**Spectral Wandering**

All fluorescent species measured in this study were observed to spectrally wander (Fig. 2a-c). Spectral wandering was quantified by analyzing single molecules that remained fluorescent for multiple frames. Briefly, the spectrum of a fluorescing single molecule in frame *n* was compared to the same fluorescing single molecule in frame *n+1* using a two sample Kolmogorov-Smirnov test to determine if there was a significant difference between the two spectra. Significantly different spectra were categorized as having spectrally wandered. The overall fractions of single molecules that spectrally wander, under the imaging conditions listed in the Methods section, are shown in Table A in S1 File. The probability of spectral wandering increased with time for all six types of probes tested here (Fig. E in S1 File). The average magnitude of the change in peak emission wavelength was calculated for the single molecules that were identified by the Kolmogorov-Smirnov test as having spectrally wandered (Table A).

**TABLE A.**

|  | Fraction of Spectral Wandering ± (S.E.M.) | Change in Wavelength (nm) |
| --- | --- | --- |
| Dendra2 | 0.133 ± 0.005 | 13.5 ± 0.2 |
| PAmCherry | 0.162 ± 0.008 | 14.2 ± 0.4 |
| PAmKate | 0.296 ± 0.030 | 12.8 ± 0.3 |
| Cage552 | 0.207 ± 0.009 | 11.1 ± 0.2 |
| Cage590 | 0.299 ± 0.009 | 10.6 ± 0.2 |
| Cage635 | 0.262 ± 0.014 | 11.0 ± 0.3 |

By summing up the spectral measurements for single molecules that both spectrally wander and fluoresced for several frames, we reconstituted the bulk fluorescence spectra of the species under investigation. Fig. F in S1 File shows a PAmCherry molecule that fluoresced for two frames (short dashes) before photobleaching. The sum of the spectral measurements (long dashes) closely resembles the bulk emission spectra measured from PAmCherry (solid line). Despite the nearly overlapping relationship of the measured and bulk spectra, several more frames would be required to fully reconstruct the spectrum of PAmCherry due to the expected broadening of the Spectral-FPALM spectrum from the contribution of diffraction (Fig. 1f). Not all molecules fluoresce long enough to fully recreate the bulk spectra; however by lowering excitation intensity and increasing the camera frame rate it may be possible to image the molecule during multiple spectral wandering events.

**Monochromator measurements**

Calibration was performed by illuminating a 0.5 µm pinhole with a narrow range of wavelengths from a monochromator. We measured the emission from the monochromator from 400 nm to 800 nm at 50 nm intervals using a spectrometer. Spectral measurements showed that the desired wavelength matched the measured wavelength to within 0.5 nm with a typical width (FWHM) of 10-12 nm (Fig. G in S1 File).

**Localization of Pinhole Image for Microscope Calibration**

The calibration for Spectral-FPALM requires precise alignment of the spectral channel with the spatial channel as a function of detection wavelength. To accomplish this alignment, the light from a monochromator is focused through a 0.5 μm pinhole which is imaged by the microscope (see Materials and Methods), and the pinhole is localized from its image. Because the pinhole is not point-like, its image is slightly larger than diffraction-limited, which is expected to cause slightly higher localization uncertainty, which would degrade the quality of spatial and spectral channel registration, except that we are also able to detect a very large number of photons (typically >5000 photons) per pinhole image. These numerous photons allow the pinhole image to be localized with a measured precision of ~5 nm (Fig. H in S1 File), which we calculate is sufficient for registration of the spatial and spectral channels to <7 nm.

Measuring greater numbers of photons from the pinhole for the purposes of calibration simply requires increasing the acquisition time per frame. A larger number of detected photons will improve the localization precision of the pinhole, which improves precision of the alignment between the spectral and spatial channels.

**Difference in Localization Precision using Two Detection Channels**

The localization precision of each single molecule is expected be slightly worse when emission is split between two detection channels compared to a single channel because the photons are divided between those two channels. Because a second channel is needed for spectral measurements, it is useful to calculate the degradation in localization precision due to the reduced number of photons. The two dimensional localization precision equation in Thompson et al 2002[^33^](#_ENREF_33) is

$\left\langle\left(\Delta x\right)^{2}\right\rangle=\frac{s^{2}+\frac{a^{2}}{12}}{N}+\frac{8\pi s^{4}b^{2}}{a^{2}N^{2}}$ Eq. 1

In the above equation, *s* is the resolution, *a* is the pixel size, *N* is the number of detected photons, and *b* is the background noise. We modify the equation to determine the localization precision in one of the two channels.

$\left\langle\left(\Delta x\right)^{2}\right\rangle_{t}=\frac{s^{2}+\frac{a^{2}}{12}}{c_{1}N}+\frac{8\pi s^{4}\left(c_{2}b^{2}\right)}{a^{2}\left(c_{1}N\right)^{2}}$ Eq. 2

The parameter *c_1_* is the fraction of the total number of photons, *N*, originating from a single molecule that contributes to the spatial localization. The fraction of the signal that contributes to the background signal reaching the spatial channel is *c_2_*_­_. In the case where the background and fluorescence signal are split with the same fraction (*c_1_=c_2_*), the localization precision reduces to

$\left\langle\left(\Delta x\right)^{2}\right\rangle_{t}=\frac{1}{c_{1}}\left\langle\left(\Delta x\right)^{2}\right\rangle$ Eq. 3

Under the above condition and assuming the beamsplitter splits 50% of the light independent of wavelength, the localization precision will be √2 worse than if all photons were detected in a single channel. In real situations, the beamsplitter transmits slightly less than 50% of the photons in the spectral region of interest and the transmission rate decreases at longer wavelengths (BS013, Thorlabs), so the localization precision will be slightly worse than √2.

This approach assumed that all imaged background originated from the sample and will be split in some fraction. However, camera readout noise contributes to the overall background noise and will not be reduced in each channel. Additional background that is not part of the optical path (such as room or equipment lighting) will also not be reduced for each channel. Background levels can be separated based on the criteria of whether they are split by the beamsplitter or not:

$b^{2}=\alpha^{2}+c_{2}\beta^{2}$ Eq. 4

In this equation, *α^2^* represents the background levels due to the camera readout or other sources not affected by the beamsplitter. The value *β^2^* represents the background levels that are split by the beamsplitter: cellular autofluorescence, the autofluorescence of any optics in the detection path, or other sources along the beampath. Eqs. 1 and 2 can be expanded into Eqs. 5 and 6, respectively, by substituting in Eq. 4:

$\left\langle\left(\Delta x\right)^{2}\right\rangle=\frac{s^{2}+\frac{a^{2}}{12}}{N}+\frac{8\pi s^{4}\alpha^{2}}{a^{2}N^{2}}+\frac{8\pi s^{4}c_{2}\beta^{2}}{a^{2}N^{2}}$ Eq. 5

$\left\langle\left(\Delta x\right)^{2}\right\rangle_{t}=\frac{s^{2}+\frac{a^{2}}{12}}{c_{1}N}+\frac{8\pi s^{4}\alpha^{2}}{a^{2}\left(c_{1}N\right)^{2}}+\frac{8\pi s^{4}\left(c_{2}\beta^{2}\right)}{a^{2}\left(c_{1}N\right)^{2}}$ Eq. 6

By combining equations 5 and 6, the relationship between the localization precision in the transmitted channel and the single channel localization precision is

$\left\langle\left(\Delta x\right)^{2}\right\rangle_{t}=\frac{1}{c_{1}}\left\langle\left(\Delta x\right)^{2}\right\rangle+\frac{8\pi s^{4}b^{2}}{a^{2}N^{2}}\left(\frac{c_{2}-c_{1}}{c_{1}^{2}}\right)$ Eq. 7

The extra term in Eq. 7 becomes negligible for larger numbers of detected photons or small values of *α* and *β*. When *c_1_*→1 and *c_2_*→1 (representing the single channel limit), the second term vanishes and the equation converges to the localization precision expected for a single channel measurement. Because *c_1_* and *c_2_* are the fractions of light and background reaching each channel respectively, this equation remains valid for more than two channels.

**Spectral measurements of background in cells**

NIH-3T3 cells were mock-transfected with Lipofectamine 2000 (Invitrogen) and fixed with 4% PFA. Spectral measurements of the background signal were carried out under typical excitation and activation intensities at 405 nm and 561 nm wavelengths. The emission spectrum was measured with a spectrometer (USB-2000, Ocean Optics, Dunedin, FL). In order to record as much of the spectrum as possible, the dichroic in the filter cube was replaced with a piece of glass containing a small (<1 mm diameter) mirror to reflect the excitation and activation lasers and all bandpass filters were removed except for 405 nm and 561 nm notch filters.

Mock-transfected NIH-3T3 cells contain a double peak in background, at ~560 nm and ~680 nm, with a considerable signal in between (Fig. J in S1 File). Removal of the peak at ~600 nm may not be feasible if probes such as Dendra2 or PAmCherry are present in the sample as such background removal would eliminate the majority of the desired signal. For the fluorescent probes used here, removal of the background at >650 nm can be achieved with a bandpass filter to reduce the total cellular background signal by ~50%.

Of primary concern is the appearance of this background on spatial images recorded in our Spectral-FPALM setup. Measurements of the mock-transfected cells indicate that there are a considerable number of single molecules activating rather than a diffuse low intensity background fluorescence of the cell. With appropriate filters to block fluorescence greater than 650 nm, the remaining background single molecules typically fall into the regime of Dendra2 or PAmCherry (Fig. B in S1 File) and account for the higher rate of misidentification in PAmKate sample (Fig. 2 f). Thus, care should be taken to choose filters that block as much background as possible while still allowing for the maximum spectral fluorescence signal to be detected from each probe species.
